# The Forgotten Voices: Enabling Children and Young People With Intellectual Disability to Express Their Views on Their Inpatient Hospital Experience

**DOI:** 10.1111/hex.70168

**Published:** 2025-02-04

**Authors:** Jo Wray, Jessica Russell, Faith Gibson, Charlotte Kenten, Kate Oulton

**Affiliations:** ^1^ Centre for Outcomes and Experience Research in Children's Health Illness and Disability (ORCHID), Great Ormond Street Hospital for Children NHS Foundation Trust London UK; ^2^ Institute of Cardiovascular Science University College London London UK; ^3^ School of Health Sciences University of Surrey Guildford Surrey UK; ^4^ Marie Curie Palliative Care Research Department University College London London UK

**Keywords:** children and young people, inpatient care, intellectual disability, patient‐reported experience measure

## Abstract

**Background:**

The importance of enabling patients to provide feedback on their experience of healthcare is widely accepted but there are few appropriate measures to enable children and young people to directly provide feedback, particularly those with intellectual disability or younger children. Our primary aim was to develop and test patient‐reported experience measures for children and young people with intellectual disability who use inpatient hospital services. A secondary aim was to test these measures with younger children aged 4–7 years without intellectual disability.

**Methods:**

A hospital inpatient patient‐reported experience measure previously developed for children aged 8–11 years was revised iteratively in conjunction with a parent advisory group (comprising five parents of children and young people with and without intellectual disability) and children and young people with intellectual disability. The final patient‐reported experience measure was tested in children's inpatient wards at seven hospitals in England over a 3‐month period.

**Results:**

Parents suggested a need for a single patient‐reported experience measure for all children with intellectual disability which contained simpler language, fewer questions and response options, and images to illustrate each question. The final patient‐reported experience measure had 22 questions, each with a corresponding image, and a free text box in which children could add anything further. Questions addressed environment, people, care and treatment and safety.

During testing at seven children's hospitals, 52 children and young people with intellectual disability (4–18 years) and 76 children without intellectual disability (4–7 years), all of whom received inpatient care, completed the patient‐reported experience measure. Two percent of data were missing; in 16 instances respondents either ticked two responses or placed a tick between two response categories. Half the respondents added comments and/or drew a picture.

**Conclusion:**

The development of a patient‐reported experience measure for children and young people with intellectual disability provides a previously unavailable opportunity for them to report on their experiences of inpatient care and have their voices heard. Future work to extend testing to a wider group is underway and will enable us to clarify whether one patient‐reported experience measure is valid and reliable for all children and young people with intellectual disability.

**Patient or Public Contribution:**

A parent advisory group, comprising parents/carers of young people with or without a learning disability, advised on all aspects of the wider study design and specifically on revisions to the measure reported in this manuscript. The parent advisory group met twice a year during the study with email contact between meetings for specific requests and study updates. Representatives of the advisory group also attended the study steering group.

**Abbreviations**
CYP: children and young people
PREM: patient‐reported experience measure

## 1 Background

The WHO defines quality of care as ‘the extent to which health care services provided to individuals and patient populations improve desired health outcomes’ [1] and one key component of quality of care is ‘experience of care’, which includes effective communication, care delivered with respect and dignity and access to appropriate emotional support [2]. The importance of enabling patients to provide feedback about all aspects of their health care is now widely accepted [3, 4]. The development of appropriate measures to ensure inclusion of all patient groups has, however, not kept pace—and in particular for children and young people (CYP). The UN Convention on the Rights of the Child, Articles 12 and 13 [5] state that children have the right to both be heard and to freedom of expression. However, whilst they may be recognised as active agents with a ‘voice’ [6], their diverse communication needs and requirement for developmentally appropriate tools to enable them to express their views are less frequently recognised.

Significant and increasing numbers of CYP access hospital care every year [7, 8], a proportion of whom have intellectual disability. Children with intellectual disability have an increased risk for more complex health needs, higher healthcare utilisation, more frequent and lengthier hospital stays and poorer experiences of care than their peers without intellectual disability [9, 10, 11]. However, they are rarely, if ever, asked about their healthcare experiences or involved in service improvements, thereby increasing the health inequities experienced by a group of patients who are already marginalised [12]. Recent NICE guidance for disabled CYP with complex needs [13] highlights that they should be encouraged and supported to ‘give their views on their health, care…and express what they want and need’. To do this they need the means by which they can meaningfully express their views and provide feedback on their hospital experience, and hospital trusts have a legal duty under the Equality Act to make necessary reasonable adjustments to facilitate this. However, a recent rapid evidence synthesis of tools and methods for measuring healthcare experiences of people with intellectual disability emphasised the lack of surveys available for this group, particularly for CYP [14]. Furthermore, as highlighted in the NHS Learning Disability Standards for England, NHS Trusts must go further to ‘involve people, families and carers in all aspects of planning and evaluating care and treatment, and use their feedback and experiences to improve services’ and ‘demonstrate that they co‐design relevant services with people with learning disabilities, autism or both and their families and carers’ [15]. Similarly, younger children (typically less than 8 years of age) are also rarely directly included in any evaluation of their health care, despite evidence that children as young as 4 years of age are able to reliably report on their experience [16].

### 1.1 Context for the Current Project

Recent years have seen the development of several Patient Reported Experience Measures (PREMs) for children, but the dominance of adult input in terms of both development and completion of PREMs is a concern and the focus of many existing PREMs has been on acute rather than chronic health conditions and secondary rather than tertiary care [17, 18, 19, 20]. To address some of these perceived gaps we developed and tested PREMs for CYP aged 8–16, focusing on both inpatient and outpatient hospital experiences, during a 2‐year project [21]. PREMs capture patients’ perspectives on the process (the ‘what’ and ‘how’) of care and are increasingly seen as indicators of healthcare quality [22, 23, 24]. We undertook a robust four‐stage process involving: (1) understanding experiences of hospitalisation to inform the content of the questionnaire; (2) questionnaire design and testing; (3) presentation testing; and (4) acceptability testing and piloting [21]. The result was a series of PREMs for three age groups (8–11, 12–13 and 14–16), with separate PREMs for inpatients and outpatients; these were also subsequently culturally adapted and translated for use in other countries [25, 26]. The driving principle was that the PREMs were developed *by* CYP *for* CYP, and, in contrast to measures developed by adults for CYP, it was only those topics identified by CYP as having an impact on their experience of care that were included in the final questionnaire. Three key themes about hospital experience were identified and included in the PREMs: facilities, treatment and tests and people working at the hospital. CYP also decided on the ‘look and feel’ of the PREMs, resulting in two different designs being chosen for the younger and older CYP. However, we also recognised that whilst we had taken important steps towards enabling CYP to express their views on their hospital experience, our PREMs were not suitable in their current form for completion by children with intellectual disability or by younger children. The existing PREMs did not have representative images and whilst they were visually engaging they relied on children being able to read and understand written information; as such they were not accessible for many children with intellectual disability or younger children.

Our primary aim of the current work was to develop and test inpatient CYP PREMs for CYP with intellectual disability. A secondary aim was to test the PREMs with younger children without intellectual disability aged 4–7 years.

## 2 Methods

As a starting point for the instrument development we took our existing CYP PREM for inpatients aged 8–11, which had established face validity and demonstrated feasibility and acceptability.^21^ The PREM comprises five questions about the respondent (e.g., gender, age) and 32 questions about their hospital experience. Response categories are a mix of 3‐, 4‐ or 5‐point scales, with children being asked to tick one option for some questions or ‘all that apply’ for other questions. Given the focus that CYP had placed on the three domains of facilities, treatment and tests, and people working in the hospital in the original piece of work (which also included CYP with intellectual disability) it was considered important to align with the original domains in the new PREM.

To adapt the existing PREM, a staged approach was used, in an iterative process of consultation and revisions to the PREM prior to testing (Table 1):

1. The PREM was discussed in detail by a parent advisory group, comprising 10 parents of CYP with and without intellectual disability who had long‐term health conditions, as part of a study addressing access to safe and high‐quality hospital care for CYP with intellectual disability. Group members were shown the existing PREM and asked to provide comments on each question, which were documented verbatim by the group facilitator, as well as feedback about response options. They were particularly asked to think about language, which questions should be prioritised and any areas that were missing.
2. Based on the feedback from the parent advisory group, the research team crafted questions for an ‘Easy‐Read’ survey.
3. Further review by the parent advisory group as part of the assessment of face validity.
4. An artist was commissioned to develop images to sit alongside each question, with a corresponding ‘thumbs up’ or ‘thumbs down’ or ‘smiley face’ or ‘sad face’ for CYP to indicate their response.
5. In‐patients aged 5 years and older with and without intellectual disability at a specialist paediatric hospital were recruited, via the hospital school, to provide feedback on the images. In 1:1 sessions they were shown the images and asked what they thought was being depicted and for any further comments. Their comments were documented.
6. The artist made minor revisions to the images in response to the feedback.
7. As part of the ‘Pay More Attention’ study [27] children at seven hospitals were invited to complete the new PREM over a 3‐month period. The local principal investigator at each hospital identified up to four wards and, to facilitate ease of distribution and collection of PREMs, all CYP with and without intellectual disability who were aged between 4 and 18 years and discharged during the data collection period were eligible to participate. Paper questionnaires were provided and were given by ward staff to CYP and their parents before they were discharged. At the start of the questionnaire, there was a tick box question for the respondent to indicate who had completed the questionnaire (child on their own, child with parent, parent). Parents were asked to indicate if their child had an intellectual disability, a long‐term condition, both or neither. Parents completed a parent PREM as part of the study, which took the form of a more standard questionnaire, and all participants were advised that return of a completed PREM was taken as their consent/assent to participate. PREMs were completed anonymously, placed in an envelope and posted in a study box on the ward before being returned to the research team by the local principal investigator. No members of the research team were directly involved in data collection.

**Table 1:** Stages of instrument revision and testing.

Stage	Actions taken
1. Review of existing survey	Presentation of existing PREM for children aged 8–11 (with established face validity and demonstrated feasibility and acceptability) to parent advisory group Comments about the content and appearance of PREM documented verbatim
2. Revision of survey	Easy read survey developed by the research team
3. Review of survey	Assessment of face validity by parent advisory group
4. Revision of survey	Artist commissioned to develop images for revised survey
5. Patient feedback	Children who were inpatients provided feedback on survey; comments recorded
6. Revision of survey	Minor revisions to images
7. Testing of final survey	CYP who were inpatients in one of seven children's hospitals during a 3‐month period were invited to complete survey Analysis of completeness of data and variation in response

In this manuscript our aim is to consider the development and feasibility of using of the PREM in real‐time across several organisations, focusing on children and young people with intellectual disability and younger children aged between 4 and 7. Data are presented on completion rates for each question for the two groups of CYP. Findings related to the development and testing of the PREM are reported in accordance with the SQUIRE reporting guidelines [28] What the data tell us about the experience of children with intellectual disability in hospital will be reported as part of a manuscript on the broader experience of children in hospital.

## 3 Results

Five out of ten parent‐advisory group members provided feedback and suggestions for the initial modification of the PREM during face‐to‐face meetings and by email.

### 3.1 Stages 1–3—Parent Advisory Group Feedback

The overall response of the parent advisory group to the existing PREM was positive and there were relatively few suggestions for revision. They suggested that, in contrast to the original PREM, there should be one survey for all CYP with intellectual disability, regardless of age. They thought the survey was too long, particularly for younger children, and they suggested that fewer questions from each section should be included, and that each question should be illustrated with relevant images or symbols. There was some discussion about using Widgit symbols to portray each question but it was decided that drawn images would be preferrable. Suggestions were also made about simplifying language and/or making it more CYP‐friendly. The group suggested that smiley faces/sad faces could be used to indicate positive or negative answers to questions. The response categories were considered to be too complex, the consensus was that there should be two response categories for each question, and the decision was then made to use ‘thumbs up/thumbs down’ response options. Table 2 indicates specific feedback in relation to individual questions. The parent advisory group also identified some further questions (Table 2) but for pragmatic reasons it was decided that additional questions should not be added, particularly as this would have made the survey longer but also, importantly, because these topics had not come from the CYP themselves.

**Table 2:** Suggestions from the parent advisory group about revising the CYP‐PREM.

Original question	Comments
‘Did staff make you feel welcome?’	Considered to be old fashioned language. Replace with ‘Were the staff friendly’
‘How long did you have to wait to see a doctor or nurse?’	Waiting is not something only done at the start of an admission—can be at any point (e.g., child waiting all day to see a doctor and doctor does not come. Child does not have question answered). Child may not know who the people coming in and out of room are. They may not know how long they had to wait, particularly if they were distracted by an activity/DVD. Remove this question.
‘How did you feel before you had any tests and treatment’	This is an important question to include
‘During your stay in hospital, did you trust the people looking after you?’	This is an important question to include
‘Do you think the hospital staff were good at their job? (Always, Sometimes, Never)’	CYP unlikely to know this: there are a number of people they will see once and never again. This question could cause anxiety. Remove this question.
‘How would you describe your overall experience of staying in hospital? (Very good, good, in between, bad, very bad)’	Too many response options.
‘Did you feel that you were ready to leave the hospital on the day you went home? (No I would like to have stayed longer, Yes I felt ready to leave the hospital)’	This will be being answered on the day they leave the hospital. This question should be removed.
‘Which of these is the main language spoken at home?’	Remove this and ask parents for this information.
Additional questions for consideration suggested by the parent advisory group	Question about care being discussed in front of CYP Do you think your mum/dad were well looked after during your stay in hospital? Did you have everything you needed? (e.g., could they access the toilet/hoists available/able to have a bath/wheelchair accessible/changing tables?) Did the doctors understand your needs?

### 3.2 Stages 4–6

**Figure 1:**
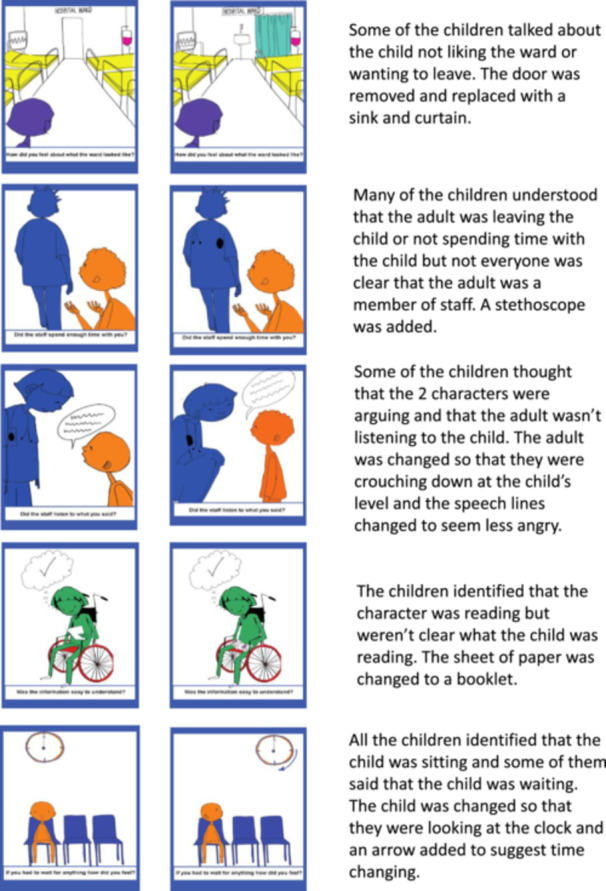
Changes to images after consultation with children and young people with and without intellectual disability.

Seven CYP (aged 6–14; five had intellectual disability) from three different inpatient wards in a specialist children's hospital provided feedback about the images. Changes were made to five images based on the feedback, as shown in Figure 1. CYP were able to articulate what the images were depicting—for example, ‘He's feeling sad’; ‘He has had to wait so long’; ‘Man is listening’ and ‘She doesn't feel like the ward looks right’.

**Figure 2:**
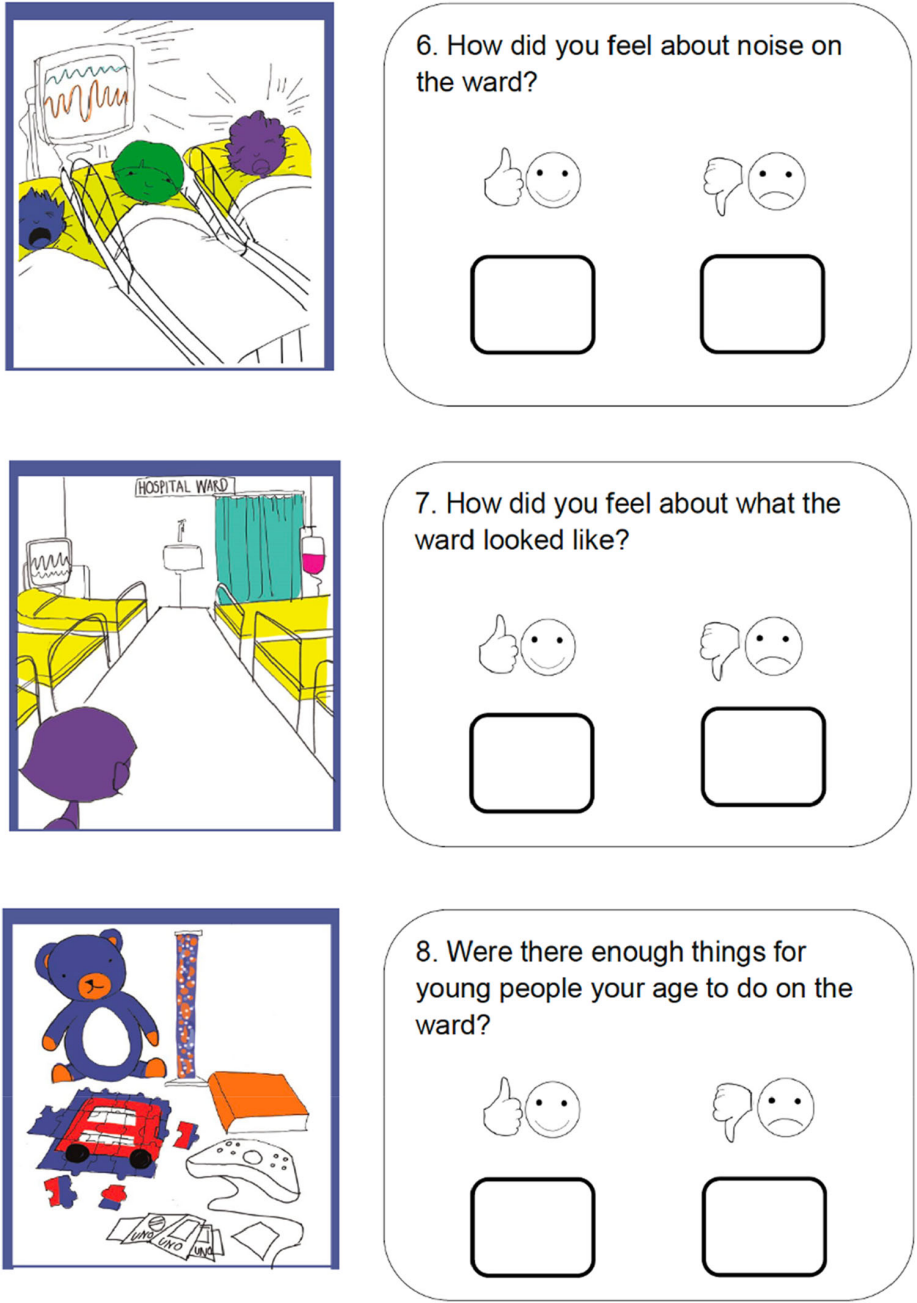
Excerpt from the final CYP‐PREM (intellectual disability).

The final PREM had 22 questions, each of which had a corresponding image, arranged over eight pages, with a free text box at the end in which children could write or draw if they had anything else they wanted to say. An excerpt from the PREM is shown in Figure 2. The first three questions asked children about their gender, whether they stayed in a bay or cubicle and whether they had stayed in hospital before. The remaining 19 questions addressed environment (*n* = 6), people (*n* = 5), care and treatment (*n* = 6), trust and safety (*n* = 2).

### 3.3 Stage 7

Completed questionnaires were received from 137 CYP aged 4 and above with intellectual disability and from 195 CYP aged 4–7 without intellectual disability. However, in 90 and 121 cases, respectively, parents had assisted their child in completing the PREM. These were excluded from any further analysis, leaving a sample of 47 CYP with intellectual disability and 74 children aged 4–7 without intellectual disability who reported having completed the PREM unaided. Characteristics of the CYP are shown in Table 3.

**Table 3:** Characteristics of the children completing the PREM.

	Children with intellectual disability, *n* (%)	Younger children, *n* (%)
Age group		
4–7 years	8 (17)	74 (100)
8–12 years	14 (30)	0 (0)
13–18 years	25 (53)	0 (0)
Gender		
Boy	27 (57)	38 (51)
Girl	20 (43)	35 (47)
Not known	0 (0)	1 (1)
Long‐term health condition		
Yes	12 (26)	16 (22)
No	35 (74)	51 (69)
Not known	0 (0)	7 (9)
Stayed in hospital before		
Yes	39 (83)	35 (47)
No	8 (17)	39 (53)
Admission planned/unplanned		
Planned	28 (53)	32 (43)
Unplanned	17 (36)	28 (38)
Not known	2 (4)	14 (19)
Place on ward where they stayed		
Cubicle	21 (45)	20 (27)
Bay	23 (49)	53 (72)
Cubicle and bay	3 (6)	1 (1)
Hospital		
1	8 (17)	3 (4)
2	4 (9)	12 (16)
3	1 (2)	2 (3)
4	8 (17)	20 (27)
5	16 (33)	13 (18)
6	6 (13)	19 (26)
7	4 (9)	5 (7)

#### 3.3.1 Completeness of Data

Table 4 shows the proportion of missing data for each question and each group. Overall, there were few missing data (47/2299; 2%). For any individual question, the highest number of missing responses was six (8%) and two (4%) for younger children and children with intellectual disability, respectively. Sixteen of the 47 ‘missing’ responses (34%) were answered with a tick in between the two response options or both were ticked; the rest were blank. One child (aged 6, no intellectual disability) had four missing responses, four had three missing responses and seven had two missing responses. Twenty‐nine (24%) CYP had one or more missing responses, 17 of whom had missed one question.

**Table 4:** Proportion of missing responses and responses to each of the experience questions for the two groups of children.

Question	Response	Children with intellectual disability, *n* (%)	Younger children, *n* (%)
Are you a girl or boy?	Missing	0 (0)	0 (0)
Have you stayed in hospital before?	Missing	0 (0)	0 (0)
When you were on the ward did you stay in your own cubicle or a bay?	Missing	0 (0)	0 (0)
How did you feel about where you stayed on the ward?	Thumbs up	44 (94)	65 (88)
	Thumbs down	2 (4)	7 (9)
	Missing	1 (2)	2 (3)
How did you feel about sleeping on the ward?	Thumbs up	36 (77)	51 (69)
	Thumbs down	9 (19)	17 (23)
	Missing	2 (4)	6 (8)
How did you feel about noise on the ward?	Thumbs up	25 (53)	45 (61)
	Thumbs down	21 (45)	28 (38)
	Missing	1 (2)	1 (1)
How did you feel about what the ward looked like?	Thumbs up	45 (96)	65 (88)
	Thumbs down	2 (4)	9 (12)
	Missing	0 (0)	1 (1)
Were there enough things for young people your age to do on the ward?	Thumbs up	36 (77)	66 (89)
	Thumbs down	10 (21)	8 (11)
	Missing	1 (2)	0 (0)
How did you feel about waiting for things in hospital?	Thumbs up	31 (66)	34 (46)
	Thumbs down	14 (30)	37 (50)
	Missing	2 (4)	3 (4)
Did you have enough privacy when staying on the ward?	Thumbs up	43 (91)	64 (86)
	Thumbs down	4 (9)	8 (11)
	Missing	0 (0)	2 (3)
Did you feel safe when staying on the ward?	Thumbs up	45 (96)	71 (96)
	Thumbs down	1 (2)	3 (4)
	Missing	1 (2)	0 (0)
Did you trust the people looking after you?	Thumbs up	46 (98)	72 (97)
	Thumbs down	1 (2)	2 (3)
	Missing	0 (0)	0 (0)
How did you feel about having any tests or treatments?	Thumbs up	34 (72)	47 (64)
	Thumbs down	12 (26)	24 (32)
	Missing	1 (2)	3 (4)
Before you had any tests or treatments, did you understand what was going to happen?	Thumbs up	38 (81)	52 (70)
	Thumbs down	8 (17)	22 (30)
	Missing	1 (2)	0 (0)
Did the staff help you if you had pain or were not comfortable?	Thumbs up	45 (96)	68 (92)
	Thumbs down	1 (2)	4 (5)
	Missing	1 (2)	2 (3)
Did the staff spend enough time with you?	Thumbs up	45 (96)	65 (88)
	Thumbs down	2 (4)	8 (11)
	Missing	0 (0)	1 (1)
Were the staff friendly?	Thumbs up	46 (98)	74 (100)
	Thumbs down	0 (0)	0 (0)
	Missing	1 (2)	0 (0)
Did the staff listen to what you said?	Thumbs up	44 (94)	71 (96)
	Thumbs down	3 (6)	2 (3)
	Missing	0 (0)	1 (1)
Did the staff do something about what you said?	Thumbs up	44 (94)	68 (92)
	Thumbs down	2 (4)	4 (5)
	Missing	1 (2)	2 (3)
While you were in hospital, did you get enough information?	Thumbs up	45 (96)	65 (88)
	Thumbs down	2 (4)	6 (8)
	Missing	0 (0)	3 (4)
Was the information easy to understand?	Thumbs up	41 (87)	57 (77)
	Thumbs down	5 (11)	15 (20)
	Missing	1 (2)	2 (3)
Did you have a say in any decisions?	Thumbs up	39 (83)	55 (74)
	Thumbs down	7 (15)	16 (22)
	Missing	1 (2)	3 (4)

#### 3.3.2 Variation in Response

**Figure 3:**
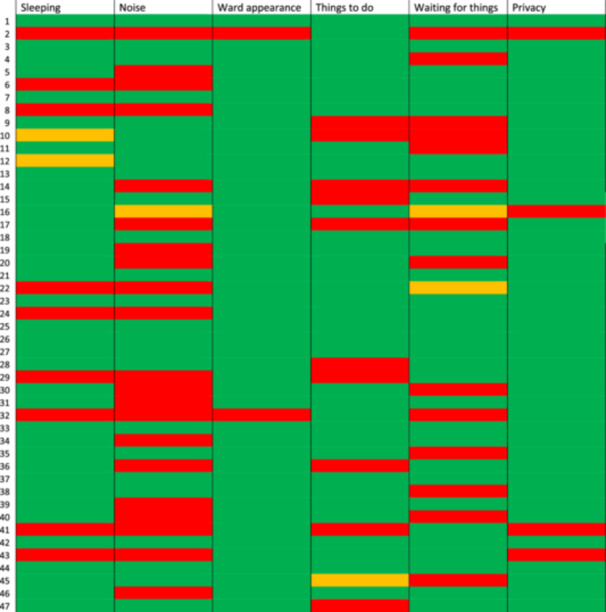
Visual Individualised Likert Data (VILD) plot illustrating the responses of the 47 CYP with intellectual disability on six questions about the environment. Each row represents an individual respondent; each column represents a different question. 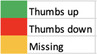.

For one question, related to the friendliness of staff, all but one CYP responded positively whereas for some other questions, such as those related to waiting, having tests and treatment and noise, the proportion of positive responses was lower (54%–67%), suggesting that CYP were able to discriminate between the questions. Thirteen younger children (18%) and eight children with intellectual disability (17%) answered all questions with the same response. Figure 3 provides an example of a VILD plot [29] showing responses for the 47 CYP with LD on six of the questions relating to the environment.

#### 3.3.3 Free Text Comments

Fifty (41%) CYP across both groups added comments and 18 (15%) CYP drew a ‘picture’ on the questionnaire, such as a smiley face, a picture of themselves with the nurse or doctor or something which was harder to decipher. Most of the comments were positive—‘thank you’, ‘you done good’, ‘the doctors were very nice and friendly…made me laugh and gave me loads of things to do’, ‘I liked the play specialist, she brought me sewing, it was fun’ but some CYP commented on specific things that they struggled with ‐ ‘bed uncomfortable’, ‘cream not working so could feel needle’, ‘noisy babies in the room’ and ‘more toys, ‘very boring in hospital’.

## 4 Discussion

We previously developed PREMs for CYP aged 8–16 years [21] but recognised that they were not inclusive—CYP with intellectual disability and those younger than 8 years were not included, thereby precluding these groups from expressing their views. To address this we took a pragmatic approach to the development of a PREM for CYP with intellectual disability/younger CYP. CYP with intellectual disability were included in the development of the initial PREM and the consensus among the wider research team from the ‘Pay More Attention’ study [27] was that we should revise, iteratively, the PREM developed for CYP aged 8–11 with engagement from parents of CYP with intellectual disability and CYP with intellectual disability themselves, thereby adhering to the important principle of defining measures of patient experience for, and validating them with, the user groups for whom they are intended [30]. Including CYP with intellectual disability in research activities can be challenging for a range of reasons—including ethical concerns and communication and cognitive difficulties [31]—but we believed that we could overcome some of these challenges by adapting an existing measure developed *by* CYP *for* CYP and which had established face validity and demonstrated feasibility and acceptability. The fact that there were few suggestions for significant revisions does, we think, speak to the rigour of our original approach in developing the PREM and its clear resonance with CYP and families. The suggestions to simplify the language into an easy read version, with supporting images, and to reduce the length of the PREM were largely anticipated. What we had not necessarily considered was that one PREM to include children with intellectual disability from 4 to 18 years of age and younger children without intellectual disability would be the preferred option. This approach highlights the importance of considering mental rather than chronological age and, on reflection having one version for all CYP with intellectual disability makes the process of administration easier and removes the need to use a value judgement about which version of a PREM a young person with intellectual disability should be asked to complete. This will likely aid uptake in clinical practice although it would still be necessary for staff administering the questionnaire to know that a child or young person has an intellectual disability that would make it difficult for them to complete the version for CYP without an intellectual disability.

Children find it difficult to process a range of choices of response options and two response options were considered most appropriate by the parent advisory group for children with intellectual disability or younger children. The trade‐offs are a loss of granularity and children struggling to answer a question where they do not agree with either response option. Although completeness of the PREM was good, with few missing data for any individual question, one‐third of the missing data were because children wanted a third option. The challenge is how to develop a solution that accommodates those for whom two options are appropriate as well as those who require more response options and this needs to be considered in the next stage of testing and validating the PREM. One option would be to include some space to write comments next to a response box although our experience has been that CYP prefer all free text comments to be together on a questionnaire and this approach would still preclude scoring an individual question. Previous findings also suggest that very young children (2–3 years old) display a bias towards answering ‘yes’ on a question with a yes/no response option [20]; potential response bias needs to be explored further with children with intellectual disability.

There are several limitations that need to be taken into consideration. First, we asked parents of CYP with and without intellectual disability to revise the existing PREM, rather than CYP with intellectual disability themselves. However, although it is widely acknowledged that parents and CYP are likely to have differing views [32], CYP, including those with intellectual disability, developed the original content and we considered that parents would be better able to advise on language and questionnaire length at this initial stage, although in future work these aspects will be explored with CYP in more detail. We did, however, consider it important that CYP were involved in providing feedback about the images which was then used to revise them. We recognise that the approach we took to revising our existing PREM does make an assumption that CYP with intellectual disability have the same perceptions, concerns and expectations as both their parents and CYP without intellectual disability and that the ideal approach would have been to develop a PREM specifically for CYP with intellectual disability and for parents’ views not to be included. As part of the larger study [27] we used other approaches such as Talking Mats and a sticker exercise to collect data from a small group of children with intellectual disability about their hospital experience. Aspects such as the hospital environment, waiting, being listened to, privacy and the friendliness of staff were all mentioned as being important. Furthermore, the free text comments collected as part of the testing reflected the questions asked in the PREM and there was nothing in them that suggested that further questions were required. Whilst we believe that the PREM captures what is important to CYP with intellectual disability, we are exploring this in further work in which we are asking CYP with intellectual disability to tell us about any gaps and redundancies in the PREM as well as exploring the use of widgets and photographs for specific groups. Although we asked participants to indicate whether the parent, parent and CYP, or CYP alone completed the PREM, it is possible that this information was not accurately completed. We are not able to calculate a response rate as we do not know how many CYP were offered the PREM to complete in each of the participating hospitals, for how many CYP the PREM was not appropriate or how many declined to complete it. We also do not know the level of intellectual impairment of the participants.

We have reported the initial stages of the development of a PREM specifically for CYP with intellectual disability/younger children and based on the completeness of the data and the fact that almost half of the children either added comments and/or drew a picture, the PREM appears to have been well received by these groups of CYP. The PREM broadly aligns with the domains in the WHO framework [2] which are specific to ‘experience of care’ for CYP and families—respect and dignity, effective communication and emotional support—but further work is now required to test the PREM with more diverse groups of both CYP with intellectual disability and younger children to enable us to assess further elements of validity and reliability and to determine whether one PREM is valid and reliable for all CYP with intellectual disability/younger children. Once this work is complete the PREM will be able to be administered routinely to CYP with intellectual disability and younger children receiving inpatient care (with similar work required to develop and test the outpatient version(s)). NHS Trusts have quality and safety teams and within our own institution we have a quality and safety strategy which includes elements that are captured in the PREM as well as a quality improvement team that works with individual hospital teams to improve the quality of care delivered to children and families. Results from other routinely collected PREMs, particularly the Friends and Family Test [33], are fed directly back to clinical teams as well as to the quality and safety team and other executive‐led hospital boards and results are also displayed in wards and outpatient areas for patients and families to see. There is a strategy for making changes in response to feedback, in conjunction with the quality improvement team if indicated, and our intention would be for findings from a routinely used PREM for CYP with intellectual disability to be used and reported in the same way.

## 5 Conclusion

The development of a PREM for CYP with intellectual disability and younger children provides a previously unavailable opportunity for them to report on their experiences of inpatient care and have their voices heard. Furthermore, the study demonstrates that these CYP are able and willing to express their views about their hospital experience. The development of the PREM is an important step towards ensuring equity in the ability of CYP to meaningfully report on their hospital experience and for their views to inform the development and delivery of children's health care in hospital settings.

## Author Contributions

**Jo Wray:** conceptualisation, funding acquisition, writing–original draft, investigation, formal analysis, writing–review and editing, methodology, data curation, supervision. **Jessica Russell:** writing–review and editing, data curation, formal analysis, methodology. **Faith Gibson:** conceptualisation, funding acquisition, writing–original draft, investigation, formal analysis, data curation, methodology, writing–review and editing. **Charlotte Kenten:** data curation, supervision, writing–review and editing, formal analysis, methodology. **Kate Oulton:** conceptualization, investigation, funding acquisition, writing–original draft, writing–review and editing, formal analysis, data curation, supervision, project administration, methodology.

## Ethics Statement

Ethical approval for the study was granted by London–Stanmore Research Ethics Committee (16/LO/0645).

## Consent

Consent for completion of the anonymous patient‐reported experience measure was assumed on return of the questionnaire.

## Conflicts of Interest

The authors declare no conflicts of interest.

## Data Availability

Questionnaire data are available on reasonable request to the authors. The data are part of a wider data set which is not publicly available at the current time as further analysis and dissemination are still to be completed.
